# An Appreciation

**Published:** 1921-06-04

**Authors:** S. S. Goldwater

**Affiliations:** The Mount Sinai Hospital, Fifth Avenue and One Hundredth Street, New York


*To the Editor of The Hospital.*

### An Appreciation.

*To the Editor of The Hospital.*

SIR,—I have read most appreciatively your kind remarks in your issue of April 30, on my recently published review of progress in hospital administration during the past year. May I take advantage of this opportunity to say how thoroughly I enjoy reading THE HOSPITAL from week to week? The way in which THE HOSPITAL handles broad questions of public health is admirable. I know of nothing in this country which is comparable to it.—Yours very truly,

S. S. GOLDWATER, M.D.

The Mount Sinai Hospital,
Fifth Avenue and One Hundredth Street,
New York, May 21, 1921.

